# Relationship of sarcopenia with steatohepatitis and advanced liver fibrosis in non-alcoholic fatty liver disease: a meta-analysis

**DOI:** 10.1186/s12876-018-0776-0

**Published:** 2018-04-19

**Authors:** Rui Yu, Qiangwei Shi, Lei Liu, Lidong Chen

**Affiliations:** 1 Department of Gastroenterology, The First Affiliated Hospital of Zhengzhou University, Zhengzhou, China; 2 Department of Cardiology, The First Affiliated Hospital of Zhengzhou University, Zhengzhou, China; 3 Department of Nasology, The First Affiliated Hospital of Zhengzhou University, Zhengzhou, China

**Keywords:** Sarcopenia, Nonalcoholic fatty liver disease, Meta-analysis, Steatohepatitis, Liver fibrosis

## Abstract

**Background:**

Several studies have emerged indicating that sarcopenia is associated with nonalcoholic fatty liver disease, we aimed to systematically review and quantify the association between sacropenia and the histological severity of nonalcoholic fatty liver disease.

**Methods:**

Pubmed, the Cochrane Library and EMBASE were searched (until August 2017) for studies examining the relationship of sarcopenia with steatohepatitis and advanced liver fibrosis in nonalcoholic fatty liver disease. Pooled odds ratios were estimated by fixed effects models.

**Results:**

Three articles met our inclusion criteria, with a total of 3226 individuals. Two of the studies examined the association between sacropenia and steatohepatitis, a significant association was documented between sarcopenia and steatohepatitis (OR = 2.35, 95%CI 1.45, 3.81). All of the three studies assessed the association between sacropenia and advanced liver fibrosis, a significant association between sarcopenia and advanced liver fibrosis (OR = 2.41, 95%CI 1.94, 2.98). No significant heterogeneity was detected among studies in all comparisons. These results remained essentially unchanged after excluding any of the studies in the sensitivity analysis.

**Conclusions:**

Sarcopenia in patients with nonalcoholic fatty liver disease is associated with a higher likelihood of having steatohepatitis or advanced liver fibrosis. Demonstration of the role of sarcopenia in nonalcoholic fatty liver disease development in future studies could have important therapeutic implications.

**Electronic supplementary material:**

The online version of this article (10.1186/s12876-018-0776-0) contains supplementary material, which is available to authorized users.

## Background

Non-alcoholic fatty liver disease (NAFLD) has become the predominant cause for chronic liver disease in the Western countries and is estimated to impact more than 30% of Americans [1] and Chinese [2], with the prevalence continuously rising. Globally, the prevalence of NAFLD is rising as a result of increasingly sedentary lifestyle, globalization of Western diet and increasing frequency of obesity [3]. NAFLD comprises of a wide spectrum of disease including simple steatosis, non-alcoholic steatohepatitis (NASH), from liver fibrosis to cirrhosis, liver failure and hepatocellular carcinoma. It is generally recognised that the presence of histologic NASH is associated with worse outcomes compared to no-NASH NAFLD and the general population [4, 5]. Several studies have shown that NAFLD with advanced fibrosis is a significant predictor of mortality from cardiovascular diseases [6] as well as of liver-related events [7]. Thus, early assessment and intervention of this high-risk subjects may reduce the burden associated with these diseases.

Sarcopenia is characterised by generalised and progressive loss of skeletal muscle mass and strength, frequently associated with poor quality of life, physical disability and death [8]. Previously, it was regarded as consequence of ageing; but recently, studies identified it as a progressive disease frequently associated with multisystem disorders [9]. As both diseases - sarcopenia and NAFLD – share similar mediator such as insulin resistance, increased inflammation and physical inactivity, many studies have emerged over the past few years examining the relationship of sarcopenia with the presence of NAFLD [10, 11] and its severity [12–15]. In our study, we aimed to systematically review and quantify the association between sacropenia and the histological severity of NAFLD. We hypothesised that sacropenia was associated with steatohepatitis and advanced liver fibrosis in non-alcoholic fatty liver disease.

## Methods

### Search strategy

Relevant studies were identified by a PubMed, EMBASE, and the Cochrane Library literature search with the following terms: (“sarcopenia” MeSH or “sarcopenia” or “sarcopenic obesity” or “muscle wasting”) and (“non-alcoholic fatty liver disease” MeSH or “fatty liver” MeSH or “non-alcoholic fatty liver disease” or “fatty liver” or “steatohepatitis” or “NASH” or “NAFLD”). Moreover, we examined the reference lists of relevant reviews and original papers. The information contained in this report is based on articles published before August 2017.

### Study selection

We developed strict criteria for categorizing the studies by two independent reviewers (QWS and RY). We included all studies that reported data on sarcopenia with steatohepatitis or liver fibrosis of NAFLD. We excluded studies that examined other types of liver disease (viral hepatitis,alcoholic liver disease, toxin-induced liver injury, hepatocellular carcinoma); studies without original data; in vitro or animal studies; and papers absent or inadequate information about sarcopenia, study population, or not enough information to calculate the odds ratio (OR) and 95% confidence intervals (CI).

### Data extraction

Data were extracted independently by two investigators (QWS and RY) and then cross-checked. When data were unclear or required assumptions to be made, a third investigators (LL) was consulted so that a consensus could be reached before recording an entry in the database. We abstracted key study characteristics of selected publications, including country, publication year, participant characteristics (age, gender, ethnicity), study design, method of diagnosis of sacropenia, NAFLD, liver steatosis and fibrosis. The quality of reporting was assessed by an evaluation system modified from the Newcastle-Ottawa Scale (NOS) [16], the meta-analysis was conducted following the Meta-analysis of Observational Studies in Epidemiology (MOOSE) guidelines [17].

### Statistical analysis

All analyses were performed using Stata software (v. 12.0; Stata, College Station, TX). *P* < 0.05 was considered significant. In all analyses, pooled ORs and 95% CIs were calculated. The significance of the pooled OR was calculated by the Z-test. A fixed effect model (Inverse Variance) was chosen in the present study. The study heterogeneity was assessed using the Cochran’s Q statistic and I^2^ statistic, considering a Q statistic *P* < 0.1 or *I*^*2*^ > 50% as significant heterogeneity. Owing to the limited number of studies included in each analysis, publication bias was not assessed.

The sensitivity analyses were also conducted to assess the consistency of results and to investigate the influence of one study on the overall meta analysis. It was carried out by sequential omission of individual studies.

## Results

### Search results

**Fig. 1:**
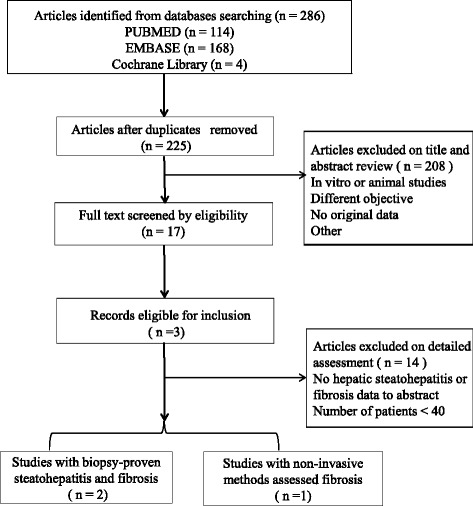
Flowchart showing the selection of articles included in the meta analysis. (by Microsoft Powerpoint)

The search strategy yielded 286 studies. Seventeen of these 286 were considered of potential value, and we retrieved full texts for further evaluation. After detailed assessment and exclusion, 3 studies were included (Fig. 1). The main characteristics of the studies analyzed are summarized in Table 1. The publication year of the studies ranged from 2016 to 2017. Of the 3 studies included, 2 rest in Asia [13, 14], one originated in Europe [12].

**Table 1:** Characteristics of studies included in meta-analysis on sarcopenia with steatohepatitis and liver fibrosis

Year	First Author	Design	NAFLD (N)/ Total (N)	Country	Age (Years)	Gender (% Male)	Method of diagnosis of sarcopenia	Method of diagnosis of NAFLD	Method of diagnosis of liver fibrosis	Study quality^1^
2017	Petta [12]	Cross-sectional	225/225	Italy	48.3	62.7	BIA	Liver biopsy	Liver biopsy	8
2017	Koo [13]	Cross-sectional	240/309	Korea	53	46.9	BIA	Liver biopsy	Liver biopsy	9
2016	Lee [15]^a^	Cross-sectional	2761/2761	Korea	55.8	55	DXA	NLFS [32]	NFS [33]	6

*BIA*, Bioelectric impedance analysis, *DXA* Dual energy X-ray absorptiometry, *LSM* liver stiffness measurement, *NLFS* NAFLD liver fat score, *NFS* The NAFLD fibrosis score

^a^NAFLD and liver fibrosis was defined using different prediction models, data of the first method was caculated and presented

^1^Methodological quality of included studies assessed using a method based in the 9-star Newcastle-Ottawa Scale

### Article characteristics

NAFLD was ascertained by liver biopsy in two studies [12, 13], and by non-invasive prediction models in one study [15]. The sarcopenia was diagnosed by the skeletal muscle mass index (SMI), which was evaluated by bioelectric impedance analysis (BIA) in two of the studies [12, 13], by dual energy X­ray absorptiometry (DXA) in one [15]. Meta-analyses involved 3226 individuals for liver fibrosis and 465 individuals for NASH. The quality of included studies according to NOS is presented in Table 1, no study was excluded on the basis of poor NOS score (Additional file 1).

### Meta-analysis

**Fig. 2:**
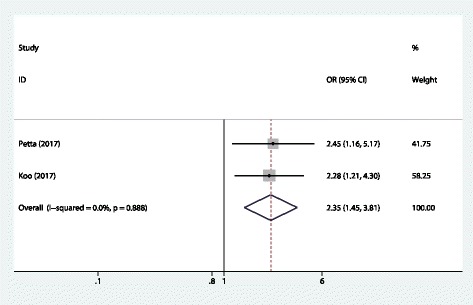
Forest plot of the meta-analysis performed to investigate the association between sarcopenia and NASH. (by Stata and Adobe Illustrator)

For NASH, two studies examined the association between sacropenia and NASH. Both two studies showed a direct relationship between sarcopenia and and NASH. Overall, we found a significant association between sarcopenia and NASH (OR = 2.35, 95%CI 1.45, 3.81). The Cochran’s Q statistic and I^2^ statistic did not show heterogeneity among the studies (*P* > 0.1; Fig.2).

**Fig. 3:**
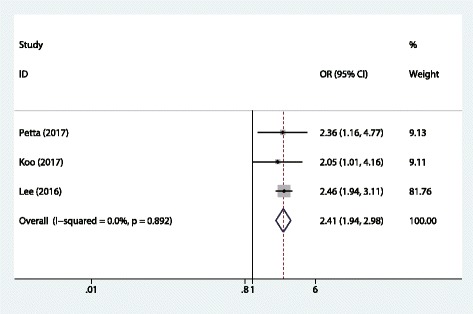
Forest plot of the meta-analysis performed to investigate the association between sarcopenia and ALF. (by Stata and Adobe Illustrator)

For liver fibrosis, a total of 3 studies examined the association between sacropenia and advanced liver fibrosis (ALF). Figure 3 shows the pooled ORs for ALF. From three studies analyzed, all of them showed a direct relationship between sarcopenia and and ALF. Overall, we found a significant association between sarcopenia and ALF (OR = 2.41, 95%CI 1.94, 2.98). The Cochran’s Q statistic and I^2^ statistic did not show heterogeneity among the studies (*P* > 0.1; Fig.3).

In the sensitivity analysis, after excluding any of the studies, there were only minimal changes in the comparisons between groups.

## Discussion

A significant direct association was found between sarcopenia and NASH (OR = 2.35, 95%CI 1.45, 3.81) or ALF (OR = 2.41, 95%CI 1.94, 2.98) in this systematic review and meta-analysis including more than 3000 NAFLD patiens. Several hypotheses could be put forward to further explain the complex inter-relationships between sarcopenia and NAFLD.

Skeletal muscle plays a key role in insulin resistance as a primary tissue contributing to whole body insulin-mediated glucose uptake [18], muscle depletion reduces the predominant cellular target for insulin action, inducing glucose intolerance and accelerating gluconeogenesis, which conversely, will promote proteolysis and muscle wasting. Moreover, insulin resistance results in lipolysis and accordingly generation of free fatty acids, which can easily be taken by both muscle and liver [19, 20]. The insulin resistance-mediated muscle depletion mass and thus the less uptake of free fatty acids by muscle will further result in exposure to and increased uptake in free fatty acids by liver [21].

Growth hormone (GH) and insulin-like growth factor-1 (IGF-1) is another potential link between sarcopenia and NAFLD. GH and IGF-I play important roles in linear growth in childhood, and continue to have essential metabolic fuctions throughout whole life. GH and IGF-1 are key effectors of changes in muscle mass. It has been well-described that abdominal and visceral obesity have strong influence on the suppression of GH secretion, and obese patients with the lowest GH secretion presenting the most severe metabolic complications; reduced GH and IGF-1 levels seen in patients with obesity, may be detrimental to both skeletal muscle and liver, contributing to ectopic fat storage [22]. Growing evidence has revealed that GH as well as IGF-I also play important roles in the liver, GH/IGF-1 axis impairment seems to be associated to the risk of both developing to sarcopenic obesity and ectopic fat storage in the liver [23–25].

Chronic inflammation and oxidative stress have been shown to prompt muscle wasting [26]. The interaction of several intracellular signaling pathways may affect the balance between protein synthesis and breakdown, inducing apoptosis, which cause the primary pathology of muscle wasting [27]. Oxidative stress and chronic inflammation also can result in a stress response of hepatocytes, leading to the development of NASH and progression of fibrosis [28].

Additionally, the link between sarcopenia and NAFLD severity may partially owing to the vitamin D deficiency frequently found in patients with liver diseases. Growing evidence has established a close association between vitamin D status and sarcopenia. Some data suggest that vitamin D deficiency may impair muscle function and is associated with sarcopenia, furthermore, low vitamin D status is associated with lower muscle mass, poorer muscle function, and predicts more severe muscle loss and disability development [29]. As well, the association between vitamin D levels and NAFLD has been increasingly recognized. A meta-analysis with seventeen studies demonstrated that NAFLD patients had significant lower vitamin D levels and higher probability of vitamin D deficiency, compared to controls [30]. In vitro study, vitamin D supplementation was shown to ameliorate NASH progression in choline-deficient and iron-supplemented l-amino acid-defined diet-induced NASH model [31], suggesting a potential role of vitamin D in the development of NAFLD.

Overall, the overlap in the pathophysiology of sarcopenia and NAFLD make it challenging to determine whether sarcopenia is a risk factor for NASH, or it is a complication of NASH. The two entities are so intricately intermeshed that the presence of either one may increases the risk for the other. Clinicians should have an increased awareness of sarcopenia to diagnose it in patients with NAFLD, and should intervene early in these high-risk patients. Further studies are needed to assess intervention in sarcopenia and the development of pharmacologic intervention that addresses both conditions.

To our knowledge, this is the first meta-analysis to investigate sarcopenia with steatohepatitis and advanced liver fibrosis in NAFLD. Another strength of this study was that only high quality articles based on NOS quality assessment were included. The limitations of the study need to be considered: (a) all studies included are of cross-section nature, thus a cause-effect relationship between sarcopenia and NAFLD severity cannot be drawn. (b) Two of the studies used liver biopsy-the gold standard, while another study used noninvasive markers to identify NAFLD cases and advanced histology, the different method for NAFLD diagnosis may result in increased risk of publication bias. (c) some of the original studies did not provide ORs adjusted for potentially important confounders, such as BMI, age, and presence of diabetes.

## Conclusions

In summary, this meta-analysis demonstrated that sarcopenia was associated with the severity of NAFLD. The possible causal relationship between sarcopenia and the severity of NAFLD, which needs to be proven by future prospective studies, may offer attractive therapeutic opportunities for treatment of NAFLD.

## Additional file

Additional file 1: **Table S1.** Methodological quality of included studies assessed using a method based in the 9-star Newcastle-Ottawa Scale. (DOCX 26 kb)

## Abbreviations

ALF: advanced liver fibrosis
BIA: bioelectric impedance analysis
CI: confidence intervals
DXA: dual energy X­ray absorptiometry
GH: growth hormone
IGF-1: insulin-like growth factor-1
NAFLD: Non-alcoholic fatty liver disease
NASH: non-alcoholic steatohepatitis
NOS: Newcastle-Ottawa Scale
OR: odds ratio
SMI: skeletal muscle mass index

## References

[1] Williams CD, Stengel J, Asike MI, Torres DM, Shaw J, Contreras M, Landt CL, Harrison SA (2011). Prevalence of nonalcoholic fatty liver disease and nonalcoholic steatohepatitis among a largely middle-aged population utilizing ultrasound and liver biopsy: a prospective study. Gastroenterology.

[2] Wong VW, Chu WC, Wong GL, Chan RS, Chim AM, Ong A, Yeung DK, Yiu KK, Chu SH, Woo J (2012). Prevalence of non-alcoholic fatty liver disease and advanced fibrosis in Hong Kong Chinese: a population study using proton-magnetic resonance spectroscopy and transient elastography. Gut.

[3] Loomba R, Sanyal AJ (2013). The global NAFLD epidemic. Nat Rev Gastroenterol Hepatol.

[4] Soderberg C, Stal P, Askling J, Glaumann H, Lindberg G, Marmur J, Hultcrantz R (2010). Decreased survival of subjects with elevated liver function tests during a 28-year follow-up. Hepatology.

[5] Ekstedt M, Franzen LE, Mathiesen UL, Thorelius L, Holmqvist M, Bodemar G, Kechagias S (2006). Long-term follow-up of patients with NAFLD and elevated liver enzymes. Hepatology.

[6] Kim D, Kim WR, Kim HJ, Therneau TM (2013). Association between noninvasive fibrosis markers and mortality among adults with nonalcoholic fatty liver disease in the United States. Hepatolog.

[7] Angulo P, Kleiner DE, Dam-Larsen S, Adams LA, Bjornsson ES, Charatcharoenwitthaya P, Mills PR, Keach JC, Lafferty HD, Stahler A (2015). Liver fibrosis, but no other histologic features, is associated with long-term outcomes of patients with nonalcoholic fatty liver disease. Gastroenterology.

[8] Cruz-Jentoft AJ, Baeyens JP, Bauer JM, Boirie Y, Cederholm T, Landi F, Martin FC, Michel JP, Rolland Y, Schneider SM (2010). Sarcopenia: European consensus on definition and diagnosis: report of the European working group on sarcopenia in older people. Age Ageing.

[9] The Lancet Diabetes Endocrinology (2014). Sarcopenia: a fate worth challenging. The Lancet Diabetes & Endocrinology.

[10] Lee YH, Jung KS, Kim SU, Yoon HJ, Yun YJ, Lee BW, Kang ES, Han KH, Lee HC, Cha BS (2015). Sarcopaenia is associated with NAFLD independently of obesity and insulin resistance: Nationwide surveys (KNHANES 2008-2011). J Hepatol.

[11] Kim HY, Kim CW, Park CH, Choi JY, Han K, Merchant AT, Park YM (2016). Low skeletal muscle mass is associated with non-alcoholic fatty liver disease in Korean adults: the fifth Korea National Health and nutrition examination survey. Hepatobiliary Pancreat Dis Int.

[12] Petta S, Ciminnisi S, Di Marco V, Cabibi D, Camma C, Licata A, Marchesini G, Craxi A (2017). Sarcopenia is associated with severe liver fibrosis in patients with non-alcoholic fatty liver disease. Aliment Pharmacol Ther.

[13] Koo BK, Kim D, Joo SK, Kim JH, Chang MS, Kim BG, Lee KL, Kim W (2017). Sarcopenia is an independent risk factor for non-alcoholic steatohepatitis and significant fibrosis. J Hepatol.

[14] Yamaguchi A, Ebinuma H, Nakamoto N, Miyake R, Shiba S, Taniki N, Wakayama Y, Chu PS, Saito H, Kanai T (2015). The association between sarcopenia and liver fibrosis progression in non-alcoholic fatty liver disease: assessment by non-invasive transient elastography and bioelectrical impedance body composition analyzer. Hepatology.

[15] Lee YH, Kim SU, Song K, Park JY, Kim DY, Ahn SH, Lee BW, Kang ES, Cha BS, Han KH (2016). Sarcopenia is associated with significant liver fibrosis independently of obesity and insulin resistance in nonalcoholic fatty liver disease: Nationwide surveys (KNHANES 2008-2011). Hepatology.

[16] Stang A (2010). Critical evaluation of the Newcastle-Ottawa scale for the assessment of the quality of nonrandomized studies in meta-analyses. Eur J Epidemiol.

[17] Stroup DF, Berlin JA, Morton SC, Olkin I, Williamson GD, Rennie D, Moher D, Becker BJ, Sipe TA, Thacker SB (2000). Meta-analysis of observational studies in epidemiology: a proposal for reporting. Meta-analysis of observational studies in epidemiology (MOOSE) group. JAMA.

[18] DeFronzo RA, Jacot E, Jequier E, Maeder E, Wahren J, Felber JP (1981). The effect of insulin on the disposal of intravenous glucose. Results from indirect calorimetry and hepatic and femoral venous catheterization. Diabetes.

[19] Jocken JW, Goossens GH, Boon H, Mason RR, Essers Y, Havekes B, Watt MJ, van Loon LJ, Blaak EE (2013). Insulin-mediated suppression of lipolysis in adipose tissue and skeletal muscle of obese type 2 diabetic men and men with normal glucose tolerance. Diabetologia.

[20] Jocken JW, Langin D, Smit E, Saris WH, Valle C, Hul GB, Holm C, Arner P, Blaak EE (2007). Adipose triglyceride lipase and hormone-sensitive lipase protein expression is decreased in the obese insulin-resistant state. J Clin Endocrinol Metab.

[21] Mayerson AB, Hundal RS, Dufour S, Lebon V, Befroy D, Cline GW, Enocksson S, Inzucchi SE, Shulman GI, Petersen KF (2002). The effects of rosiglitazone on insulin sensitivity, lipolysis, and hepatic and skeletal muscle triglyceride content in patients with type 2 diabetes. Diabetes.

[22] Berryman DE, Glad CA, List EO, Johannsson G (2013). The GH/IGF-1 axis in obesity: pathophysiology and therapeutic considerations. Nat Rev Endocrinol.

[23] Takahashi Y (2012). Essential roles of growth hormone (GH) and insulin-like growth factor-I (IGF-I) in the liver. Endocr J.

[24] Koehler E, Swain J, Sanderson S, Krishnan A, Watt K, Charlton M (2012). Growth hormone, dehydroepiandrosterone and adiponectin levels in non-alcoholic steatohepatitis: an endocrine signature for advanced fibrosis in obese patients. Liver Int.

[25] Poggiogalle E, Lubrano C, Gnessi L, Mariani S, Lenzi A, Donini LM (2016). Fatty liver index associates with relative sarcopenia and GH/ IGF- 1 status in obese subjects. PLoS One.

[26] Phillips T, Leeuwenburgh C (2005). Muscle fiber specific apoptosis and TNF-alpha signaling in sarcopenia are attenuated by life-long calorie restriction. FASEB J.

[27] Meng SJ, Yu LJ (2010). Oxidative stress, molecular inflammation and sarcopenia. Int J Mol Sci.

[28] Tilg H, Moschen AR (2010). Evolution of inflammation in nonalcoholic fatty liver disease: the multiple parallel hits hypothesis. Hepatology.

[29] Lappe JM, Binkley N (2015). Vitamin D and Sarcopenia/Falls. J Clin Densitom.

[30] Eliades M, Spyrou E, Agrawal N, Lazo M, Brancati FL, Potter JJ, Koteish AA, Clark JM, Guallar E, Hernaez R (2013). Meta-analysis: vitamin D and non-alcoholic fatty liver disease. Aliment Pharmacol Ther.

[31] Nakano T, Cheng YF, Lai CY, Hsu LW, Chang YC, Deng JY, Huang YZ, Honda H, Chen KD, Wang CC (2011). Impact of artificial sunlight therapy on the progress of non-alcoholic fatty liver disease in rats. J Hepatol.

[32] Kotronen A, Peltonen M, Hakkarainen A, Sevastianova K, Bergholm R, Johansson LM, Lundbom N, Rissanen A, Ridderstrale M, Groop L (2009). Prediction of non-alcoholic fatty liver disease and liver fat using metabolic and genetic factors. Gastroenterology.

[33] Angulo P, Hui JM, Marchesini G, Bugianesi E, George J, Farrell GC, Enders F, Saksena S, Burt AD, Bida JP (2007). The NAFLD fibrosis score: a noninvasive system that identifies liver fibrosis in patients with NAFLD. Hepatology.

