# Supplementary material for: Relationship of sarcopenia with steatohepatitis and advanced liver fibrosis in non-alcoholic fatty liver disease: a meta-analysis
Source: BMC Gastroenterol. 2018 Apr 19;18:51. doi: 10.1186/s12876-018-0776-0 (PMC5907709; doi:10.1186/s12876-018-0776-0)
Supplement: Supplementary file 1 — Table S1. Methodological quality of included studies assessed using a method based in the 9-star Newcastle-Ottawa Scale. (DOCX 26 kb) [file 12876_2018_776_MOESM1_ESM.docx]

**Table S1** Methodological quality of included studies assessed using a method based in the 9-star Newcastle-Ottawa Scale

| Year | First author | selection | | | | Comparability^b^ | exposure or outcome | Total |
| --- | --- | --- | --- | --- | --- | --- | --- | --- |
|  |  | Study design | Inclusion and exclusion criteria | Number of participants | Method of ascertainment of NAFLD^a^ | adjusted OR |  |  |
| 2017 | Petta[[1](#_ENREF_1)] | * | * | * | * | ** | ** | 8 |
| 2017 | Koo[[2](#_ENREF_2)] | * | * | * | * | *** | ** | 9 |
| 2016 | Lee[[3](#_ENREF_3)] | * | * | * | - | * | ** | 6 |

1. Petta S, Ciminnisi S, Di Marco V, Cabibi D, Camma C, Licata A, Marchesini G, Craxi A: **Sarcopenia is associated with severe liver fibrosis in patients with non-alcoholic fatty liver disease**. *Alimentary pharmacology & therapeutics* 2017, **45**(4):510-518.

2. Koo BK, Kim D, Joo SK, Kim JH, Chang MS, Kim BG, Lee KL, Kim W: **Sarcopenia is an independent risk factor for non-alcoholic steatohepatitis and significant fibrosis**. *Journal of hepatology* 2017, **66**(1):123-131.

3. Lee YH, Kim SU, Song K, Park JY, Kim DY, Ahn SH, Lee BW, Kang ES, Cha BS, Han KH: **Sarcopenia is associated with significant liver fibrosis independently of obesity and insulin resistance in nonalcoholic fatty liver disease: Nationwide surveys (KNHANES 2008-2011)**. *Hepatology (Baltimore, Md)* 2016, **63**(3):776-786.
